# Genetic variation of *Fusarium oxysporum* isolates forming fumonisin B_1_ and moniliformin

**DOI:** 10.1007/s13353-012-0087-z

**Published:** 2012-02-25

**Authors:** Lidia Irzykowska, Jan Bocianowski, Agnieszka Waśkiewicz, Zbigniew Weber, Zbigniew Karolewski, Piotr Goliński, Marian Kostecki, Witold Irzykowski

**Affiliations:** 1 Department of Phytopathology, Poznan University of Life Sciences, Dąbrowskiego 159, 60-594 Poznań, Poland; 2 Department of Mathematical and Statistical Methods, Poznan University of Life Sciences, Wojska Polskiego 28, 60-637 Poznan, Poland; 3 Department of Chemistry, Poznan University of Life Sciences, Wojska Polskiego 75, 60-625 Poznań, Poland; 4 Institute of Plant Genetics Polish Academy of Science, Strzeszyńska 34, 60-479 Poznań, Poland

**Keywords:** *Fusarium oxysporum*, Fumonisin B_1_, Moniliformin, Mating type idiomorphs, Genetic fingerprinting, HPLC analysis

## Abstract

Thirty single-spore isolates of a toxigenic fungus, *Fusarium oxysporum*, were isolated from asparagus spears and identified by species-specific polymerase chain reaction (PCR) and translation elongation factor 1-*α* (TEF) sequence analysis. In the examined sets of *F. oxysporum* isolates, the DNA sequences of mating type genes (*MAT*) were identified. The distribution of *MAT* idiomorph may suggest that *MAT1-2* is a predominant mating type in the *F. oxysporum* population. *F. oxysporum* is mainly recognised as a producer of moniliformin—the highly toxic secondary metabolite. Moniliformin content was determined by high-performance liquid chromatography (HPLC) analysis in the range 0.05–1,007.47 μg g^−1^ (mean 115.93 μg g^−1^) but, also, fumonisin B_1_ was detected, in the concentration range 0.01–0.91 μg g^−1^ (mean 0.19 μg g^−1^). There was no association between mating types and the mycotoxins biosynthesis level. Additionally, a significant intra-species genetic diversity was revealed and molecular markers associated with toxins biosynthesis were identified.

## Introduction

Asparagus (*Asparagus officinalis* L.) is a worldwide well-known vegetable not only due to its high nutritional value, but also because of the biologically active compounds present, including protodioscin and rutin, with a strong cytotoxic activity against many cancer cell lines (Kinghorn et al. 2003; Chin and Garrison 2008). However, the asparagus is often infected by fungi of the *Fusarium* genus, a causal agent of crown and root rot which decreases the quantity and quality of the yield. A visible chlorosis and wilting of the individual stalks, progressive discolouration of plants and destruction of roots and crown are the main symptoms of this serious disease (Logrieco et al. 2003). *F. oxysporum* Schlechtend. emend. Snyder & Hansen and *F. proliferatum* (Matsus.) Nirenberg are the most severe asparagus pathogens (Blok and Bollen 1995; von Bargen et al. 2009). Both species are well-known mycotoxins producers, so their precise identification is of prime concern. New molecular methods have been applied to complement, support and confirm identification based on morphological characters (Geiser et al. 2004).

*F. oxysporum* is identified worldwide and is known to be phylogenetically diverse and recognised as a moniliformin (MON) producer (Logrieco et al. 2003). Recent studies suggest that some *F. oxysporum* strains can also biosynthesise fumonisin B_1_-FB_1_ (Waśkiewicz et al. 2010a; Stępień et al. 2011a, b). MON exhibits cytotoxic and cardiotoxic activity, causes developmental disorders and may also induce the development of Keshan disease, human myocardial impairment reported in rural areas of China and South Africa (Pineda-Valdes and Bullerman 2000). Fumonisins are polyketide-derived mycotoxins inhibiting a ceramide synthase, the key enzyme in the sphingolipid biosynthetic pathway, resulting in serious mycotoxicoses (Soriano and Dragacci 2004; Voss et al. 2007). Contamination of food with FB_1_ has been associated with an oesophageal cancer of humans in Africa, China and the United States (Nicholson 2004). Considering an available toxicological evidence, the International Agency for Research on Cancer (IARC) describe FB_1_ as a probable carcinogenic to humans (IARC 2002). Food processing does not solve the problem because the hydro-thermal stability of fumonisin is high and decomposition products are more toxic than fumonisin itself (Bullerman et al. 2002).

*F. oxysporum* is a large taxonomic unit, described as a species complex. So far, no known sexual stage of the *F. oxysporum* is available and the concept of a biological species and the sources of genetic variation are still discussed (Kistler 1997; O’Donnell et al. 2009; Kück and Pöggeler 2009). In most fungi, the mating type locus conferring mating behaviour consists of dissimilar DNA sequences (idiomorphs) in the mating partners (Pöggeler 2001). In heterothallic species, the mating type is controlled by a single locus with two idiomorphic alleles, designated *MAT1-1* and *MAT1-2* (Kerényi et al. 1999; Turgeon and Yoder 2000). Each *MAT* idiomorph carries one gene encoding a single MAT-specific DNA binding protein. These proteins probably play a crucial role in the pathways of cell speciation and sexual morphogenesis as regulatory transcription factors. The two *MAT* alleles contain a conserved alpha (ALPHA) box domain or a high mobility group (HMG) box domain, respectively (Yun et al. 2000). Evolutionary theory indicates that sexual reproduction plays an important role in a pathogen’s evolution, i.e. the development of a new pathogenic race compatible with a disease-resistant cultivar and of a new strain resistant to a fungicide. Recently, it was shown that the presence of the *MAT1-2* idiomorph in the *F. culmorum* genome significantly affects mycelium growth (unpublished data). Assessing the possibility of mating by toxigenic strains is important for the design of successful control strategies, since these strategies are different for clonally and sexually reproducing organisms (McDonald and McDermott 1993). Moreover, there is poor information on the toxicity of pathogenic *F. oxysporum* isolates with different mating type alleles.

The present study focused on the genetic and toxigenic differentiation of *F. oxysporum* isolates. The principal aims of the study were to estimate mycotoxins biosynthesis yield and to detect mating type idiomorphs in the *F. oxysporum* genome. Additionally, the relationship between these features was assessed. Moreover, the level of intra-species genetic diversity were estimated and associations between molecular markers and mycotoxins (FB_1_ and MON) formation were examined.

## Materials and methods

### Fungal isolates

Fungi were collected from green (O-02, O-11, O-30) and white (remaining isolates) spears of asparagus ‘Gijnlim’ and ‘Eposs’ cultivated in Western Poland. Fungi were isolated from the basal part of spears (one spear per plant) on the borderline between discoloured and healthy tissues. After disinfection with 1% sodium hypochlorite, five sections (about 1 mm in diameter) of tissue were cut from spears and such a set sections was transferred onto a Petri dish with potato dextrose agar (PDA) medium. One of these fungal cultures from spears was randomly chosen for further work. Thirty single-spore cultures of *F. oxysporum* were used in the chemical and molecular analyses. All fungal isolates (O-01–O-30) were identified on the basis of their morphology (Booth 1971; Gerlach and Nirenberg 1982; Kwasna et al. 1991). The morphological features of the identified isolates were compared with standard isolates DSM-62287 (Deutsche Sammlung von Mikroorganismen und Zellkulturen, Germany) of *F. oxysporum* f. sp. *asparagi*. Isolates of DSM-62287 in this study were designated as O-31.

### DNA preparations and molecular identification of *F. oxysporum* isolates by PCR and sequencing procedures

Mycelia from 9-day-old single-spore cultures of *F. oxysporum* grown on liquid medium (5 g L^−1^ of glucose, 1 g L^−1^ of yeast extract) were separated from solution by vacuum filtration with a Büchner funnel. DNA was extracted and purified using a DNeasy Mini Kit (QIAGEN Inc., Hilden, Germany), according to the manufacturer’s recommendations. Polymerase chain reaction (PCR) with species-specific primers designed based on a partial sequence of calmodulin gene (Mulè et al. 2004a, b) was used to confirm the morphological identification of each fungal isolate. For the PCR analysis, a forward primer CLOX1: 5′-CAGCAAAGCATCAGACCACTATAACTC-3′ and a reverse primer CLOX2: 5′-CTTGTCAGTAACTGGACGTTGGTACT-3′ (Sigma, Pampisford, UK) were used. The amplification reactions were performed with a *Taq* PCR Core Kit (QIAGEN, Inc., Hilden, Germany). The reaction mixture and amplification conditions were as described previously (Waśkiewicz et al. 2010b). Amplification was carried out in a Biometra T*personal* 48 thermocycler (Whatman Biometra, Goettingen, Germany).

Additionally, for 12 representative isolates (forming the highest and the lowest amount of FB_1_ and MON), a part of the translation elongation factor 1-*α* (TEF) was amplified with the following primer pair: EF1-forward: 3′-ATGGGTAAGGARGACAAGAC-5′ (O’Donnell et al. 1998) and TEF_WI-reverse: 3′- GCTCACGRGTCTGGCCATCCTTG′ (designed in this study). Primer TEF_WI is located about 90 nt downstream of the sequence of the reverse primer designed by O’Donnell et al. (1998). The reaction mixture contained: 20 ng of fungal DNA, 0.2 mM of each dNTP, 1 μM of each primer and 0.5 U of DreamTaq™ polymerase (Fermentas GMBH, St. Leon-Rot, Germany) in 1× reaction buffer with 2 mM MgCl_2_. Amplification was carried out using the following programme: an initial denaturation for 2 min at 94°C, followed by 45 cycles of denaturation at 94°C for 30 s, primer annealing at 58°C for 30 s and extension at 72°C for 1 min. The amplification was ended with an additional extension at 72°C for 5 min. Amplification products were cleaned and then sequenced on ABI PRISM® 310 Analyzer (Applied Biosystems), according to the procedure described previously (Irzykowska et al. 2005). The resulting sequences were checked manually and were aligned using the computer software package CLUSTAL_X (Thompson et al. 1997). The obtained TEF sequences were compared with all the sequences deposited at the Fusarium ID Database (http://www.fusariumdb.org/index.php).

### Diagnostic PCR for mating type detection

To determine the mating type of the *F. oxysporum* isolates, conserved portions of the ALPHA or HMG boxes of the *MAT1-1* and *MAT1-2* idiomorphs were amplified with degenerate oligonucleotide primer pairs: forward (ALPHA) 5′-CGCCCTCTKAAYGSCTTCATG-3′ and reverse (ALPHA) 5′-GGARTARACYTTAGCAATYAGGGC-3′ for *MAT1-1* and forward (HMG) 5′-CGACCTCCCAAYGCYTACAT-3′ and reverse (HMG) 5′-TGGGCGGTACTGGTARTCRGG-3′ for *MAT1-2* (Kerényi et al. 2004). The PCRs were carried out using a *Taq* PCR Core Kit (QIAGEN, Inc., Hilden, Germany) with a reaction mixture containing: 5 ng of fungal DNA, 0.2 mM of each dNTP, 0.5 μM of each primer and 0.5 U of *Taq* DNA polymerase in 1× reaction buffer with 2.5 mM magnesium chloride. The PCR profile was as described previously (Irzykowska and Kosiada 2011).

### Genetic fingerprinting of *F. oxysporum*

Random amplified polymorphic DNA PCRs (RAPD-PCRs) were carried out using a *Taq* PCR Core Kit (QIAGEN, Inc., Hilden, Germany). The reaction mixture was as described previously (Irzykowska and Bocianowski 2008). Eight random 10-mer and one 11-mer primers, OPB-07, OPC-02, OPC-04, OPC-08, OPC-15, OPJ-10 + A, OPL-11, OPL-12 and OPL-19 (Qiagen Operon, Cologne, Germany), were used to screen the isolates for DNA polymorphism. Amplification was carried out using the following programme: an initial denaturation for 2 min at 94°C, followed by 40 cycles of denaturation at 94°C for 30 s, primer annealing at 37°C for 1 min and extension at 72°C for 2 min. The amplification was ended with an additional extension at 72°C for 5 min. PCR was repeated twice in order to check reproducibility. The comparison of each band profile for each primer was performed on the basis of the presence (1) versus the absence (0) of RAPD products of the same length.

### Electrophoresis conditions

The PCR products were separated by electrophoresis (4 V cm^−1^) in 1.5% agarose gels with 1× TBE buffer (89 mM Tris-borate and 2 mM EDTA, pH 8.0) and visualised under UV light following ethidium bromide staining. A Gene Ruler™ 100 bp DNA Ladder Plus (Fermentas GMBH, St. Leon-Rot, Germany) was used as a molecular size standard for PCR products.

### Fungal cultures for mycotoxin estimation

For toxin quantification, rice cultures were prepared for individual *F. oxysporum* isolates. Long-grain white rice (RANI) tested as being mycotoxin-free was used as a medium for the formation of mycotoxins by *F. oxysporum* isolates. Rice samples of 65 g were soaked for 16 h with 81 ml of distilled water in 500-ml Erlenmeyer flasks and then sterilised for 15 min at 120°C. Such samples were inoculated with 5-mm disks of PDA medium overgrown by *F. oxysporum* mycelium. On rice substrate, fungal cultures were incubated for 3 weeks at 20°C. All samples for analyses were prepared and cultured in triplicate (three Erlenmeyer flasks with the prepared medium) and the mean values of the results were calculated.

### Mycotoxin analyses

#### Chemicals and reagents

Standards of pure FB_1_ and MON were purchased from Sigma (St. Louis, MO, USA). Acetonitrile, methanol (high-performance liquid chromatography [HPLC] grade), disodium tetraborate, 2-mercaptoethanol and *t*-butyl-ammonium hydroxide were purchased from Sigma-Aldrich. Sodium dihydrogen phosphate, acetic acid, *n*-hexane, *o*-phosphoric acid and dichloromethane were purchased from POCh (Gliwice, Poland). Water for the HPLC mobile phase was purified using a Milli-Q system (Milipore, Bedford, MA, USA).

#### Fumonisin B_1_ analysis

Samples (10 g) of dry 3-week-old cultures were homogenised for 3 min in 20 ml of methanol–water (3:1, v/v) and filtered through Whatman no. 4 filter paper, in accordance with the method described by Sydenham et al. (1990). The detailed procedure of the extraction and purification of FB_1_ has been reported previously (Waśkiewicz et al. 2010a). Purified FB_1_ was quantitatively determined by the HPLC method. The *o*-phosphoric acid (OPA) reagent (20 mg per 0.5 ml methanol) was prepared and diluted with 2.5 ml of 0.1 M disodium tetraborate (Na_2_B_4_O_7_ × 10H_2_O) and then combined with 25 ml of 2-mercaptoethanol added to the solution. The FB_1_ standard (5 μl) or extracts (20 μl) were derivatised with 20 or 80 μl of the OPA reagent. After 3 min, the reaction mixture (10 ml) was injected onto an HPLC column. Methanol–sodium dihydrogen phosphate (0.1 M in water) solution (77:23, v/v), adjusted to pH 3.35 with *o*-phosphoric acid, after filtration through a 0.45-mm Waters HV membrane, was used as the mobile phase with a flow rate of 0.6 ml min^−1^. A Waters 2695 apparatus (Waters Division of Millipore, Milford, MA, USA), with a C-18 Nova Pak column (3.9 × 150 mm) and a Waters 2475 fluorescence detector (λ_ex_ = 335 nm, λ_em_ = 440 nm), was used in the metabolite quantitative determination. The identification and quantification of FB_1_ was observed by comparison of the retention times and peak areas in the samples with those observed for FB_1_ standard and using the relevant calibration curve (correlation coefficients for FB_1_ was 0.9967). The limit of detection (LOD) was 10 ng g^−1^ for FB_1_ and was defined as the concentration that was three times higher than the standard deviation of the blank signal. Recovery for FB_1_ measured by the mycotoxins extraction from blank samples spiked with 10–100 ng g^−1^ of the compound was equal to 93%. The relative standard deviations (RSDs) were lower than 8%.

#### Moniliformin analysis

Culture samples (15 g) of each strain were homogenised with 75 ml of acetonitrile–methanol–water (16:3:1, v/v/v) and filtered (Whatman no. 4 filter paper). Moniliformin was extracted and purified according to the methods described by Waśkiewicz et al. (2010b). MON was quantified using a Waters 501 apparatus (Waters Division of Millipore) with a C-18 Nova Pak column (3.9 × 300 mm) and a Waters 486 UV detector (λ_max_ = 229 nm). Acetonitrile–water (15:85, v/v) buffered with 10 ml of 0.1 M K_2_HPO_4_ in 40% *t*-butyl-ammonium hydroxide in 1 L of solvent (Sharman et al. 1991) was used as the mobile phase (flow rate = 0.6 ml min^−1^). The MON detection limit was 25 ng g^−1^. Positive results (on the basis of retention time) were confirmed by the HPLC analysis and by comparison with the relevant calibration curve (the correlation coefficient for MON was 0.9990). The recovery for MON was 90%. The RSD was below 7%.

### Statistical analysis

The one-way analysis of variance (ANOVA) of *F. oxysporum* occurrence was carried out in order to determine the differentiation of isolates regarding the concentrations of FB_1_ and MON. Tukey’s least significant differences (LSDs) for each trait were calculated. Homogeneous groups (not significantly different from each other) for the analysed traits were determined on the basis of least significant differences. The relationship between FB_1_ and MON concentrations was estimated using correlation coefficients (Kozak et al. 2010).

The coefficients of genetic similarity (*S*) of the investigated isolates were calculated using the following formula (Nei and Li 1979):

$$ {S_{{ij}}} = \frac{{2{N_{{ij}}}}}{{\left( {{N_i} + {N_j}} \right)}} $$

where *N*_*ij*_ is the number of alleles present at the *i*-th and *j*-th isolates, *N*_*i*_ is the number of alleles present at the *i*-th isolate, *N*_*j*_ is the number of alleles present at the *j*-th isolate, and *i*, *j* = 1, 2,…, 31. On the basis of the calculated coefficients, isolates were grouped hierarchically using the unweighted pair group method of arithmetic means (UPGMA). The relationship among isolates was presented in the form of a dendrogram (Kumar et al. 2004).

The association between RAPD markers and the FB_1_ and MON concentrations of *F. oxysporum* isolates was estimated by regression analysis (Bocianowski and Seidler-Łożykowska 2012). The molecular marker observations were treated as independent variables and considered in individual models. The relationship between *MAT* idiomorphs and the biosynthesis of FB_1_ and MON were performed by the *t*-test (Snedecor and Cochran 1989). All statistical analyses were performed using the package GenStat v.7.1. (Payne et al. 2003).

## Results

### Identification of *F. oxysporum* isolates

All of the tested isolates displayed morphology typical for *F. oxysporum*, quite similar to standard *F. oxysporum* f. sp. *asparagi* isolates DSM-62287 (here, O-31). Species-specific PCR assay targeting the calmodulin gene confirmed morphological species identification. Specific PCR products obtained for each isolate, including the reference one, was 534 bp in length.

Additionally, for representative isolates producing and non-producing mycotoxins, a 810-bp fragment of the TEF gene was sequenced. The sequences were compared with sequences deposited in the Fusarium ID database. The comparison with the TEF sequence in the Fusarium ID database showed 100% matching of all isolates examined with *F. oxysporum*, confirming the species identity (Table 1).

**Table 1:** Isolates displaying the highest and lowest toxin concentrations with 100% sequence matching with *Fusarium oxysporum* from the Fusarium ID database

Isolate	FB_1_ (μg g^−1^)	MON (μg g^−1^)	Fusarium ID isolates with 100% homology in the TEF sequence	Source of Fusarium ID isolates
O-08	0.5	60.2	FD_00056 (*F. oxysporum*)	CBS 175.35
O-10	0.9	16.1	FD_00705 (*F. oxysporum*)	ICMP 5234
O-12	0.5	9.8	FD_00056 (*F. oxysporum*)	CBS 175.35
O-15	0.3	1,007.5	FD_00805 (*F. oxysporum*)	ICMP 15895
O-17	0.0	0.6	FD_00446 (*F. oxysporum*)	CBS 151.27
O-18	0.0	0.5	FD_00446 (*F. oxysporum*)	CBS 151.27
O-21	0.0	0.0	FD_00070 (*F. oxysporum*)	CBS171.31
O-25	0.0	2.4	FD_00805 (*F. oxysporum*)	ICMP 15895
O-27	0.2	0.0	FD_00705 (*F. oxysporum*)	ICMP 5234
O-28	0.0	0.0	FD_00802 (*F. oxysporum*)	FRC O-1890
O-29	0.0	171.6	FD_00805 (*F. oxysporum*)	ICMP 15895
O-30	0.2	182.8	FD_00705 (*F. oxysporum*)	ICMP 5234

### Evaluation of mycotoxins level

HPLC analysis revealed that the majority of *F. oxysporum* isolates produce both MON and FB_1_. The results of one-way ANOVA indicate that isolates differ significantly in their toxigenic activity and mycotoxin profile (Table 2). FB_1_ was determined in the concentration range 0.01–0.91 μg g^−1^ (mean 0.19 μg g^−1^), while MON was detected in the range 0.5–1,007.47 μg g^−1^ (mean 115.93 μg g^−1^). Fumonisin B_1_ and moniliformin were formed in the highest concentrations by isolates O-10 and O-15, respectively (Table 3). Reference isolate (O-31 = DSM-62287) produced only FB_1_ at a concentration of 0.2 μg g^−1^. The FB_1_ biosynthesis level allowed to distinguish between four homogeneous groups among 31 isolates (Table 3). Two homogeneous groups (first group: O-15 isolate; second group: other isolates) were distinguished based on differences in the MON level (Table 3). Only four isolates (O-05, O-23, O-24 and O-26) did not form mycotoxins. The correlation between FB_1_ and MON was not statistically significant (*r* = 0.113, *P* = 0.544).

**Table 2:** Mean squares from the analysis of variance (ANOVA) for FB_1_ and MON concentrations (μg g^−1^) for *F. oxysporum* isolates of asparagus spears origin

Source of variation	Degrees of freedom	Biosynthesis of toxins	
		FB_1_	MON
Isolates	30	0.133***	101,576***
Residual	62	0.027	6,174

***Statistically significant (*P* < 0.001)

**Table 3:** The average values, standard deviations and homogeneous groups for FB_1_ and MON concentrations (μg g^−1^) for 31 *F. oxysporum* isolates with different mating types

Isolate code	Mating type idiomorph	FB_1_		MON	
		Mean*	Standard deviation	Mean*	Standard deviation
O-01	*MAT1-2*	0.27bcd	0.18	0.00b	0.00
O-02	*MAT1-2*	0.12bcd	0.04	0.00b	0.00
O-03	*MAT1-2*	0.06bcd	0.06	35.73b	19.98
O-04	*MAT1-1*	0.06bcd	0.05	32.60b	20.27
O-05	*MAT1-2*	0.00d	0.00	0.00b	0.00
O-06	*MAT1-2*	0.07bcd	0.04	2.86b	2.24
O-07	*MAT1-1*	0.49abc	0.37	0.00b	0.00
O-08	*MAT1-2*	0.52ab	0.29	60.23b	34.55
O-09	*MAT1-2*	0.23bcd	0.12	0.00b	0.00
O-10	*MAT1-2*	0.91a	0.28	16.07b	11.11
O-11	*MAT1-2*	0.11bcd	0.07	0.00b	0.00
O-12	*MAT1-2*	0.52ab	0.29	9.82b	4.58
O-13	*MAT1-1*	0.10bcd	0.07	105.63b	86.21
O-14	*MAT1-2*	0.30bcd	0.44	49.00b	27.10
O-15	*MAT1-2*	0.31bcd	0.21	1,007.47a	400.69
O-16	*MAT1-2*	0.06bcd	0.04	174.00b	127.06
O-17	*MAT1-2*	0.00d	0.00	0.62b	0.60
O-18	*MAT1-2*	0.01d	0.01	0.50b	0.33
O-19	*MAT1-2*	0.01d	0.01	114.03b	38.99
O-20	*MAT1-1*	0.03cd	0.03	0.00b	0.00
O-21	*MAT1-1*	0.04cd	0.04	0.00b	0.00
O-22	*MAT1-2*	0.28bcd	0.17	5.43b	4.20
O-23	*MAT1-2*	0.00d	0.00	0.00b	0.00
O-24	*MAT1-1*	0.00d	0.00	0.00b	0.00
O-25	*MAT1-1*	0.01d	0.02	2.37b	2.46
O-26	*MAT1-1*	0.00d	0.00	0.00b	0.00
O-27	*MAT1-2*	0.2bcd	0.02	0.00b	0.00
O-28	*MAT1-1*	0.03cd	0.04	0.00b	0.00
O-29	*MAT1-2*	0.01d	0.01	171.57b	53.31
O-30	*MAT1-1*	0.23bcd	0.36	182.80b	1.65
O-31	*MAT1-2*	0.02cd	0.01	0.00b	0.00
LSD_0.001_		0.47		217.8	

*Mean values followed by the same letters are not significantly different. Each letter indicates a single homogenous group

### Mating type assessment by PCR

**Fig. 1:**
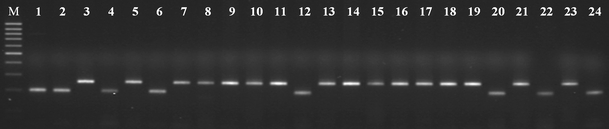
Polymerase chain reaction (PCR) amplification of mating type specific sequences from the selected *Fusarium oxysporum* isolates. Lanes: 1 (O-04), 2 (O-07), 4 (O-13), 6 (O-20), 12 (O-21), 20 (O-24), 22 (O-25), 24 (O-26): amplicon of *MAT1-1* specific ALPHA box (200 bp). Lanes: 3 (O-01), 5 (O-02), 7 (O-03), 8 (O-05), 9 (O-06), 10 (O-08), 11 (O-09), 13 (O-10), 14 (O-11), 15 (O-12), 16 (O-14), 17 (O-15), 18 (O-16), 19 (O-17), 21 (O-18), 23 (O-19): amplicon of *MAT1-2* specific HMG box (260 bp). Lane: M: a Gene Ruler™ 100 bp DNA Ladder Plus (Fermentas)

In the examined set of *F. oxysporum* isolates, DNA sequences encoding *MAT1-1* or *MAT1-2* genes were detected (Fig. 1). Isolates O-04, O-07, O-13, O-20, O-21, O-24, O-25, O-26, O-28 and O-30 for which a 200-bp fragment was amplified with ALPHA primers were designated the *MAT1-1* type. The remaining 21 isolates for which a 260-bp PCR product was amplified with HMG primer pairs were designated *MAT1-2*.

The relationships between *MAT1-1* and mycotoxins biosynthesis were not statistically significant (*t* = −1.11, *P* = 0.275 and *t* =−0.65, *P* = 0.524 for FB_1_ and MON concentrations, respectively). Similarly, the correlation between *MAT1-2* and toxins biosynthesis was not statistically significant (*t* = 1.11, *P* = 0.275 and *t* = 0.65, *P* = 0.524 for FB_1_ and MON concentrations, respectively).

### Genetic variability determination

**Fig. 2:**
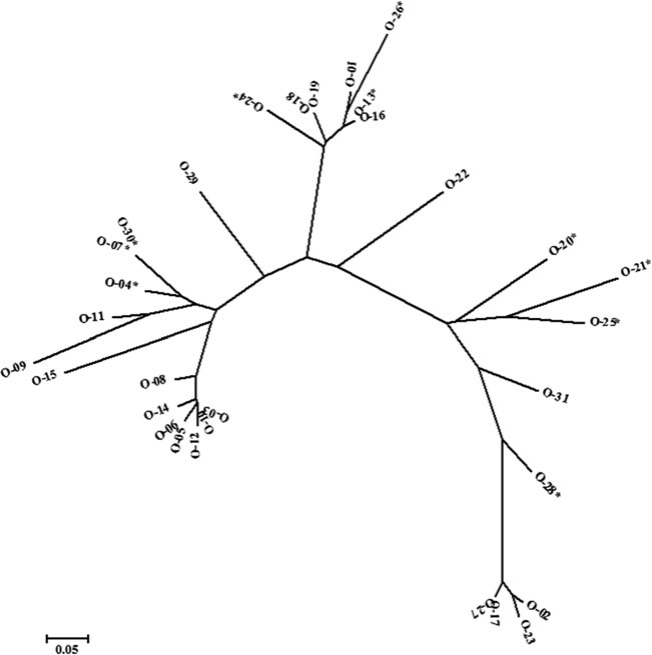
Dendrogram of *F. oxysporum* isolates based on random amplified polymorphic DNA (RAPD) data analysis; the isolates were grouped hierarchically using the unweighted pair group method of arithmetic means (UPGMA). *Isolates with *MAT1-1* idiomorph

To assess a genetic variation among *F. oxysporum* isolates, genetic fingerprinting by RAPD analyses with nine arbitrary primers was carried out. Three of them were excluded from further analysis because of monomorphism or low repeatability. Finally, 33 polymorphic products were obtained, ranging from 4 to 7 per primer (Table 4). The size of the RAPD fragments ranged from 0.15 to 3.2 kbp. The relationship among isolates of *F. oxysporum* is presented in the form of a dendrogram (Fig. 2). Clustering analysis resolved two groups at the 30% similarity level. Each of these groups was divided into smaller subgroups at 60 and 52% similarity levels, respectively. The highest genetic similarity (equal to 1) was observed between the isolates O-03 and O-10, O-7 and O-30, O-13 and O-16, O-17 and O-27, and O-18 and O-19, whereas the lowest genetic similarity (equal to 0.0667) was found for O-02 and O-12. The average value of genetic similarity was equal to 0.507.

**Table 4:** Codes and sequences of random amplified polymorphic DNA (RAPD) primers tested, with polymorphic RAPD marker numbers and sizes

Primer code	Sequence 5′-3′	Polymorphic band number	Polymorphic band size (bp)
OPB-07	GGTGACGCAG	7	a-720, b-880, c-1000, d-1470, e-2280, f-2400, g-3200
OPC-02	GTGAGGCGTC	7	a-390, b-800, c-980, d-1300, e-1460, f-1500, g-2600
OPC-04	CCGTATCTAC	–	Rejected
OPC-08	TGGACCGGTG	0	Monomorphic
OPC-15	GACGGATCAG	4	a-850, b-1150, c-1500, d-1850
OPJ-10 + A	AAGCCCGAGGA	4	a-660, b-1250, c-1370, d-2630
OPL-11	ACGATGAGCC	5	a-340, b-500, c-600, d-810, e-2010
OPL-12	GGGCGGTACT	6	a-700, b-910, c-1000, d-1130, e-1380, f-1840
OPL-19	GAGTGGTGAC	–	Rejected

**Fig. 3:**
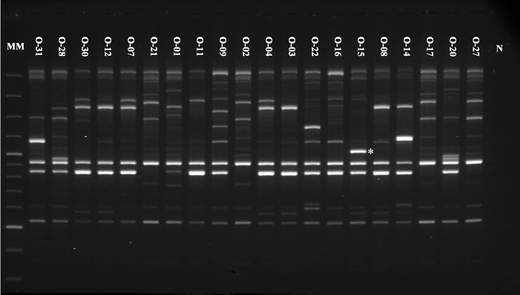
Genetic fingerprinting of *F. oxysporum* isolates applying RAPD-PCR with the OPC-15 primer. Lanes: MM a Gene Ruler™ 100 bp DNA Ladder Plus (Fermentas), N a negative control. *RAPD marker (OPC-15b) associated with high moniliformin level

Significant associations of eight markers (OPC-02b, OPC-15 d, OPL-11c, OPL-11 d, OPL-11e, OPL-12 d, OPL-12f and OPB-07b) with FB_1_ biosynthesis (Table 5) as well as one marker (OPC-15b) with MON production were found (Fig. 3). The percentage variation of FB_1_ concentration accounted by markers ranged from 11.2% (for OPB-07b) to 26.5% (for OPL-11c). In the case of MON concentration, the percentage variation accounted by OPC-15b marker was equal to 56.8%, which makes this marker a good candidate for further studies.

**Table 5:** Molecular markers significantly (at the α = 0.05 level) associated with FB_1_ concentration estimated by regression analysis

Marker symbol	Estimates of regression coefficients	*P*-value	Percentage variation accounted	Standard error of observations
OPC-02b	−0.223	0.003	24.8	0.183
OPC-15 d	0.173	0.023	13.7	0.196
OPL-11c	0.226	0.002	26.5	0.181
OPL-11 d	−0.235	0.005	22.0	0.186
OPL-11e	0.237	0.003	24.4	0.183
OPL-12 d	0.197	0.008	19.2	0.190
OPL-12f	0.184	0.012	16.9	0.192
OPB-07b	−0.167	0.037	11.2	0.199

## Discussion

When considering *Fusarium* fungi as mycotoxin producers, a proper identification of species infecting food and/or feed plants is very important. Traditional diagnostics based on morphology as well as simple PCR in some cases is insufficient. The translation factor 1-*α* gene has high phylogenetic utility because it is very informative at the species level and non-orthologous copies of them have not been detected in the *Fusarium* genus (Geiser et al. 2004). Based on species-specific PCR and partial sequencing of the TEF gene, we proved undoubtedly that isolates forming FB_1_ belong to *F. oxysporum* species.

The present study showed explicitly that some of the *F. oxysporum* isolates infecting asparagus spears have the potential to form and accumulate FB_1_, although, in general, they produced low amounts of fumonisin and significantly higher amounts of MON. The ability of *F. oxysporum* to form fumonisin has been questioned previously. Wang et al. (2010) reported that *F. oxysporum* strains isolated from asparagus spears in Zhejiang Province of China did not produce FB_1_, but only four *F. oxysporum* isolates were examined. Similarly, it was shown that *F. oxysporum* isolated from greenhouse-grown jimsonweed produced only MON at a concentration level of 3.5 μg g^−1^ (Abbas et al. 1992). On the other hand, Abbas et al. (1995) claimed that three of six isolates of *F. oxysporum* and seven isolates of *F. oxysporum* var. *redolens* from root lesions of eastern white pine seedlings produced FB_1_, ranging from traces (≥100) to 300.60 ng g^−1^. This statement was also supported by our earlier studies (Waśkiewicz et al. 2009).

Fumonisin formation is positively related to the *FUM* genes expression (Waalwijk et al. 2004; Stępień et al. 2011a, b). The *FUM* genes cluster is highly collinear among *F. oxysporum*, *F. proliferatum* and *F. verticillioides*, regarding gene number, orientation and order (Proctor et al. 2008). The amount of FB_1_ produced in vitro depends on the substrate, genotype and conditions (Desjardins et al. 1992). It was shown that there is a significant impact of interacting environmental factors on the *FUM* transcript levels (Jurado et al. 2008). The correlation between FB_1_ and MON levels was not revealed, probably because of the different biosynthetic pathways of these secondary metabolites (Voss et al. 2007). Naturally occurring genetic variance in the fungal population may explain why non-producing isolates were also recorded. The loss of toxigenic ability can occur due to even a single-point mutation that shifts the open reading frame or creates a premature stop codon (Proctor et al. 2006).

The detection of *MAT* genes in the genomes of mitosporic species is a first step into learning what causes asexuality (Kück and Pöggeler 2009). The obtained results confirm that *F. oxysporum* has a heterothallic origin and are in agreement with Turgeon’s hypothesis that some *Fusarium* species may exhibit a cryptic sexual cycle (Turgeon 1998). Similar results were reported by Arie et al. (2000) for *F. oxysporum* isolates and by Kerényi et al. (2004) for several *Fusarium* species. The amplification of the *MAT* idiomorph sequence revealed that 30% of the examined *F. oxysporum* isolates possess the *MAT1-1* idiomorph and as many as 70% possess the *MAT1-2* allele. Similar results for mating types distribution in the fungal population were described for *F. oxysporum* originated from common bean (Karimian et al. 2010). The maximum effective reproductive strategy occurs when the mating type idiomorphs are present in a 1:1 ratio (Britz et al. 1998). In many *Fusarium* species, the ratio of *MAT* idiomorphs was significantly different from the theoretical 1:1 ratio expected in an idealised population, i.e. in a population of *F. verticillioides*, the mating ratio was 2:1 for isolates originated from maize in Italy, Brazil and the Philippines (Cumagun 2007; de Oliveira Rocha et al. 2011; Venturini et al. 2011). The same mating ratio was observed for an *F. subglutinans* population in South Africa (Britz et al. 1998). Among *F. culmorum* isolates originated from wheat and rye, the mating ratio was estimated as 2:3 (Irzykowska and Kosiada 2011). The achieved results may suggest that *MAT1-2* is a predominant mating type in the *F. oxysporum* population in Poland, although a larger population of fungus originated from different geographic locations should be analysed before reaching a final conclusion. The predominance of one mating type can cause a limitation or lack of the possibility of sexual reproduction inside the population (Venturini et al. 2011). RT-PCR analysis proved that the *F. oxysporum MAT* genes are expressed (Yun et al. 2000). Lack of sexual reproduction may also be a result of the functional disorder of the yet-unidentified genes that are involved in successful sexual reproduction (Arie et al. 2000). Alternatively, asexual *Fusarium* spp. may require environmental conditions for mating that are uncommon when disease epidemics occur (Kerényi et al. 2004). To summarise, the molecular discrimination of *MAT1-1* and *MAT1-2* in *F. oxysporum* populations make possible the recognition of potentially compatible isolates that could be used in future experiments revealing whether the species is truly asexual or not.

The genetic variability of *F. oxysporum* isolates was estimated previously by different molecular methods. Genetic fingerprinting by RAPD-PCR is an effective method for determining an inter- and intra-species genetic variation without prior knowledge of the genome sequence. RAPD has been used successfully to analyse the genetic variation of several *Fusarium* species, including *F. oxysporum* (Assigbetse et al. 1994; Clark et al. 1998; Werner and Irzykowska 2007; Karimian et al. 2010), *F. proliferatum* (von Bargen et al. 2009), *F. culmorum* (Miedaner et al. 2004; Irzykowska and Baturo 2008), *F. graminearum* (Ouellet and Seifert 1993; Carter et al. 2002), *F. moniliforme*, *F. solani* and *F. avenaceum* (Khalil et al. 2003). In the present study, a significant genetic variation among *F. oxysporum* isolates was revealed and shown in the form of a dendrogram. Clustering analysis resolved several fingerprint groups, six of which contained one isolate. This is consistent with the results of another study on the genetic diversity of *F. oxysporum*, where from 6 to 10 fingerprint groups were found (Cramer et al. 2003; Zanotti et al. 2006; Karimian et al. 2010). The DNA sequences of the most unlike isolates (O-02 and O-12) differ in analysed genome parts by almost 70%. Isolate O-02 originated from green spears of asparagus ‘Gijnlim’, whereas isolate O-12 originated from white spears of asparagus ‘Eposs’, thus, it is possible that the revealed variation between them resulted from race existence. However, we have found five pairs of isolates which were identical in the examined genome parts. All of these isolates originated from white spears of asparagus ‘Eposs’. Such high genetic similarity can also be the result of the relatively low number of markers used in the study.

It is well known that a high level of intra-species genetic diversity is typical for species undergoing sexual cycle with the meiotic recombination (McDonald 1997; Kerényi et al. 2004). The ways available for genetic change in *F. oxysporum* are still largely unknown, but some possibilities exist beyond simple sexual or clonal reproduction (Kistler 1997). Daboussi and Langin (1994) reported that active fungal transposable elements may comprise up to 5% of the *F. oxysporum* genome and may have an impact on gene structure and function. Other molecular studies suggest a genetic duplication in the rDNA regions (O’Donnell and Cigelnik 1997).

Moreover, in this study, nine molecular markers associated with MON or FB_1_ formation levels were found (Table 4). The obtained PCR products will be used in future for the preliminary discrimination of toxigenic isolates. RAPD marker OPC-15b (∼1,150 bp), connected with MON biosynthesis, will be cloned and sequenced to converse them to the specific and more universal SCAR marker. Amplified regions of the *F. oxysporum* genome may be involved in the study of toxin biosynthesis pathways. A genetic background of toxin biosynthesis is complex and, probably, some genes still remain unknown. Toxicity as the majority of significant physiological traits (i.e. toxin or protein biosynthesis) is inherited quantitatively, so continuous variation arises from the segregation of alleles at many interacting loci, whose effects depend on the environment (Edwards et al. 1987; Irzykowska et al. 2001; Bocianowski and Krajewski 2009). Proctor et al. (2008) reported that the *FUM* cluster is located in different genome parts in *F. oxysporum*, *F. proliferatum* and *F. verticillioides*. Perhaps the genetic bases of FB_1_ formation by *F. oxysporum* strains have not yet been fully elucidated.

In conclusion, we postulate that *F. oxysporum* present in asparagus spears produces not only MON but also highly toxic FB_1_, which can be a significant concern for consumers. The mechanism of genetic changes occurring in *F. oxysporum* populations and the connection between the toxin profile and the genetic variability of fungus demand further attention. More extensive studies are necessary in order to elucidate the distribution of MATs in the fungus population.
